# Post-COVID-19 Recurrent Oropharyngeal Lesions Mediated by Atypical Vasculitis, an Autoimmune Condition, or Infection: A Case Report

**DOI:** 10.7759/cureus.83950

**Published:** 2025-05-12

**Authors:** Sohaib Eladl, Mohamed F Ahmed, Hesham Badr, Sara Shehab, Portia N Mzezewa

**Affiliations:** 1 Department of Accident and Emergency, Blackpool Teaching Hospitals NHS Foundation Trust, Blackpool, GBR; 2 Department of Diabetes and Endocrinology, Blackpool Teaching Hospitals NHS Foundation Trust, Blackpool, GBR; 3 Department of Respiratory Medicine, Department of Otorhinolaryngology, Blackpool Teaching Hospitals NHS Foundation Trust, Blackpool, GBR; 4 Department of Internal Medicine, Blackpool Teaching Hospitals NHS Foundation Trust, Blackpool, GBR; 5 Department of Respiratory Medicine, Blackpool Teaching Hospitals NHS Foundation Trust, Preston, GBR

**Keywords:** atypical vasculitis, covid-19 mediated iga vasculitis, covid complications, kawasaki-like disease, post covid-19 vasculitis

## Abstract

Post-COVID-19 complications have been widely identified since the pandemic began in 2019. Among these complications are immune-mediated conditions, including vasculitis, Kawasaki-like disease, and IgA vasculitis. Typically, vasculitis presents with classical features such as rash, shortness of breath, joint problems, visual problems, fibrinoid necrosis, and granulomas.

Here, we present a rare and complex case of atypical vasculitis, immunological response, or an autoimmune condition, which posed significant diagnostic challenges and required input from a multidisciplinary team (MDT).* Although the literature includes cases of COVID-19-associated complications, the combination of presentation symptoms and findings in this case is not well-documented in the literature.*

Our patient was a 46-year-old female who presented to the emergency department on multiple occasions starting in 2021 after contracting COVID-19 for nearly two months. She presented again in 2023 and 2024 with similar episodes. Despite these multiple presentations, *her* *diagnosis remained unclear for five years* until she presented in 2024 with left neck pain, progressive dysphagia, and hemoptysis.

The case was further complicated by systemic involvement as she developed small bilateral lower lung lobe peripheral emboli with no evidence of DVT or any other thrombus during her prolonged hospital stay. Biopsies and laboratory investigations were inconclusive, often showing fungal growth (Candida albicans and glabrata) and excluding malignancy.

After extensive discussions, the case was deemed to represent atypical vasculitis, an autoimmune condition, or an infection with a *very rare and complex presentation*. This case highlights that recurrent oropharyngeal and pharyngeal lesions following COVID-19 infection could indicate these possibilities, even in the absence of classical symptoms.

## Introduction

Post-COVID-19 complications pose significant clinical challenges, including vasculitis, chronic inflammation, and immune dysregulation. We presented a rare case of a 46-year-old female who developed recurrent oropharyngeal and pharyngeal lesions following a COVID-19 infection in 2021. Despite systemic involvement, the above presentation, and a positive c-ANCA (anti-proteinase 3), classic vasculitic features such as fibrinoid necrosis and granulomas were absent, resulting in a diagnostic delay of over five years.

Although a systematic review of case reports and case series in *Annals of Medicine and Surgery* highlighted various presentations of post-COVID-19 vasculitis in 2022, none matched this peculiar presentation [1]. This case highlights the diagnostic and therapeutic challenges of post-COVID-19 immune complications and the value of multidisciplinary team (MDT) management.

## Case presentation

Clinical history

A 46-year-old female presented to the emergency department in 2024 with a four-month history of left-sided neck pain and progressive dysphagia, which had worsened over 10 months to the point where she had become unable to tolerate solids or liquids. Her medical history included hiatus hernia and anxiety disorder. In 2021, she contracted COVID-19, and her symptoms began with the development of a pharyngeal mass.

In 2021, her symptoms included hemoptysis, dysphagia, and difficulty breathing. At that time, she did not experience any specific symptoms that would suggest vasculitis. Following biopsies, her symptoms were initially attributed to invasive candidiasis following COVID-19 infection, and she was treated with voriconazole. She remained stable until 2023, when she presented again to the emergency department with worsening tightness around the neck for one month and odynophagia.

**Figure 1:**
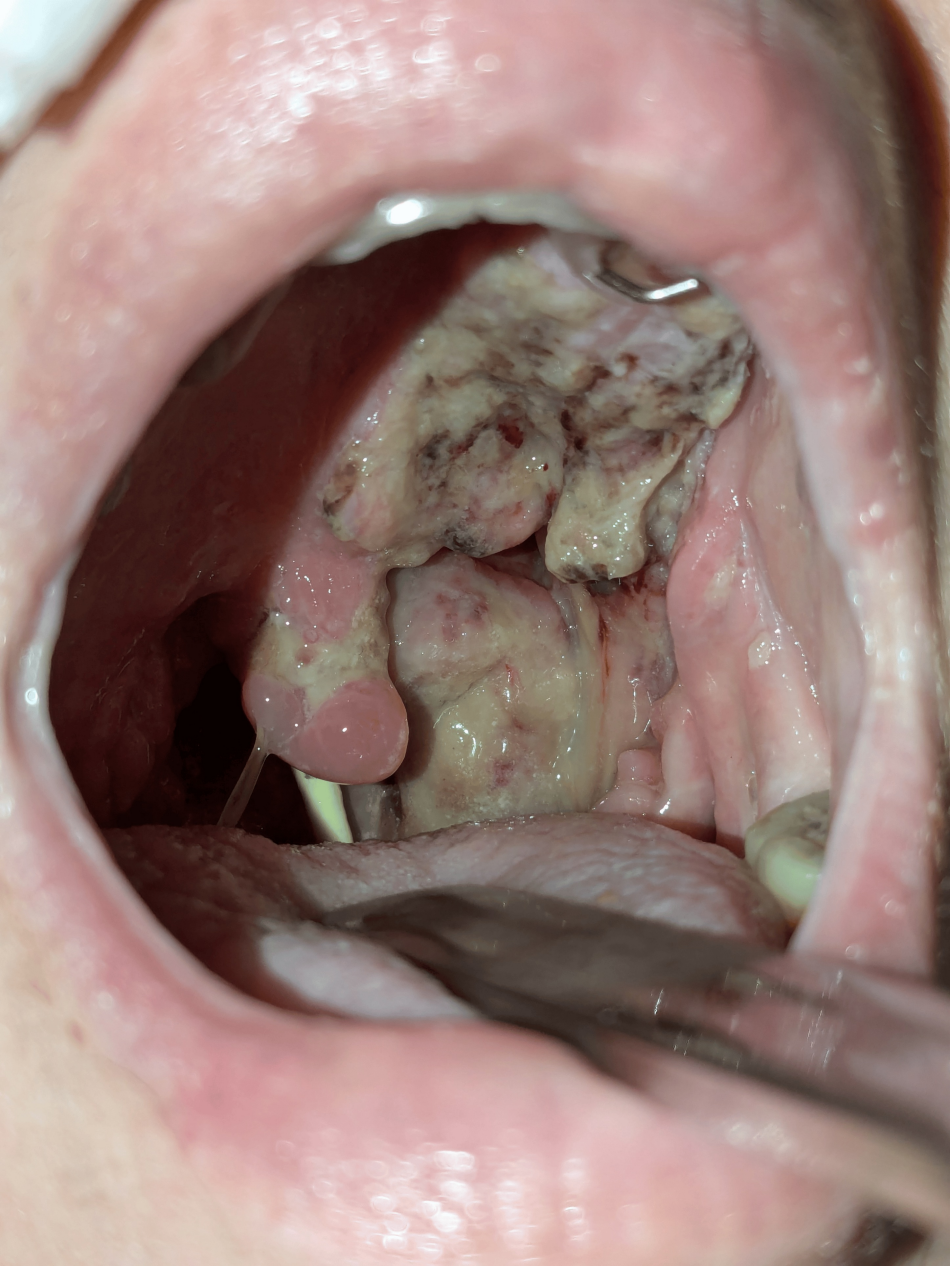
Oropharyngeal lesions.

On examination, she had mouth ulcerations and a swollen posterior pharyngeal wall with no exudates (Figure 1). She did not have any constitutional symptoms like fever, sweats, or weight loss. Biopsies were performed, which showed fungal infections with no malignancy or vasculitis evidence, and she was *discharged without a specific diagnosis, *with follow-up scheduled.

Earlier in 2024, she presented again with significant pharyngeal bleeding with no known underlying cause, similar to the previous episode in 2021 following her COVID infection. The bleeding was extensive enough to require left carotid artery ligation with tonsillar packing to control the bleeding, and she was discharged again with no clear diagnosis.

Later in 2024, she was admitted once more with identical symptoms. During this admission, she experienced acute pharyngeal hemorrhage and tonsillar bleeding, necessitating intubation. She was admitted to the critical care unit (ITU) for two days and was then extubated. Due to the extensive bleeding and deformities of the pharynx, she underwent reconstruction surgery, which was successful. She was unable to swallow for some time, requiring nasogastric (NG) feeding. Videofluoroscopy then showed deterioration of swallowing secondary to the bleeding, with no other significant findings. When the ENT team reviewed her case, they started her on dexamethasone for airway edema and requested a new biopsy, which showed neither malignancy nor a clear cause of the condition.

After a prolonged hospital stay, she developed a bilateral pulmonary embolism (PE), requiring anticoagulation with heparin infusion. The vasculitis MDT was convened, and it was concluded that overall, uncertainty remained regarding immune-mediated disease with vasculitis versus recurrent infection with potential underlying immune deficiency due to low IgG levels (3.6 g/L). The MDT also noted that her presentation was less typical of small vessel vasculitis, and biopsies described it as *"*No evidence of atypia or malignancy. No evidence of vasculitis." The vasculitis MDT recommended further investigations, including immunoglobulins, an immune deficiency panel, serum electrophoresis, IgG-4 level, and syphilis serology. Most of her remission occurred spontaneously, despite a trial of voriconazole, which the multidisciplinary team did not believe contributed to her improvement.

Radiological studies included an ultrasound of the neck, which showed nothing significant, and a CT chest, which revealed worsening lung appearance with nodularity. Diagnoses considered were atypical vasculitis versus recurrent infection with underlying immune deficiency, supported by the positive c-ANCA (anti-proteinase 3) and p-ANCA (anti-myeloperoxidase; Table 1).

**Table 1:** Blood markers. Blood markers showing positive c-ANCA (anti-proteinase 3) and p-ANCA (anti-myeloperoxidase). MPO, myeloperoxidase

Test	2024	2024	2023
Aspergillus precipitins	-	-	-
Spec. IgG to Aspergillus fumigatus	-	Negative	-
Quantitation	-	33	-
IgE to Aspergillus fumigatus	-	Negative	-
HLA B27	-	-	-
Tissue typing comment	-	-	-
Cardiolipin antibody (IgG)	0.7	-	-
Cardiolipin antibody (IgM)	<0.8	-	-
Total IgE	-	-	-
Anti-myeloperoxidase (p-ANCA)	<0.2	<0.2	0.2
ANCA screen (fluorescence)	Positive p-ANCA, not MPO. Significance unknown	Indeterminate staining pattern	Positive p-ANCA
Anti-proteinase 3 (c-ANCA)	0.7	1.7	2.6
Rheumatoid factor-latex screen	<9.75	<9.75	-

Multiple biopsies of the pharyngeal and oral lesions showed ulcerated and inflamed mucosa with prominent vasculature and granulation tissue. There was no evidence of atypia, malignancy, or fungal infection. The histopathological features were consistent with a florid reactive inflammatory process (Table 2).

**Table 2:** Tonsillar and palate biopsy.

	2024 Updated	2023 Updated
Specimen and site	Left tonsil tissue with rupture.	Biopsy palate.
Clinical details	Very unusual presentation - left tonsil / lateral pharyngeal wall acutely swollen then ruptured with haemorrhage (see previous histology from pharynx after similar presentation circa 2021, no malignancy) - Underlying vasculitis (previous necrosis of tongue and nasal septal perforation)	Carcinoma, Wegener's granulomatosis
Macro	The pot labeled left tonsil biopsy. Multiple fragments of haemorrhagic tissue measuring 15 mm x 18 mm x 8 mm in aggregate.	The pot labeled biopsy palate. Several fragmented, pale pieces of tissue ranging from 2 mm up to the largest piece, which measures 10 mm x 7 mm.
Micro	Sections show fragments of oropharyngeal mucosa with underlying skeletal muscle. There are areas of haemorrhage and necrosis with irregular scanty to moderately severe mixed inflammatory exudate. There is no lymphoid tissue in this material. There is only a small fragment of lining epithelium with no atypia. There is no evidence of vasculitis. Grocott and D-pas stains for fungi are negative.	Sections show fragments of ulcerated mucosa with irregularly distributed moderate to severe chronic inflammatory exudate. The debris on the surface contains numerous colony-forming fungal hyphae and spores. There is no evidence of atypia or malignancy. There are also no histological features of Wegener's granulomatosis.
Diagnosis	Left tonsil: Please see text	Palate: Ulceration and fungal infection reported

Differential diagnosis included infection, immunological response, or autoimmune etiology.

## Discussion

The case represents a challenging diagnosis that required multiple inputs from multiple teams due to the wide range of differentials, interventions, and diagnostic considerations. The main diagnostic considerations were post-COVID immune dysregulation, as COVID-19 is known to cause immune-mediated disorders such as vasculitis and Kawasaki-like disease. The timing of the patient’s symptoms, which began shortly after contracting COVID-19, supports this conclusion. Atypical vasculitis remains the strongest possibility, as biopsies consistently excluded atypia and malignancy, but revealed ulcerative inflammation with significant vasculature, suggesting vasculitic disease.

However, the absence of classic features such as fibrinoid necrosis and limited response to steroids makes this an uncommon presentation. Chronic invasive fungal infection was also considered, as Candida albicans was identified in some tests, but the recurrent and progressive nature of symptoms despite antifungal treatment suggests a secondary process. Immune deficiency was another diagnostic consideration due to her low IgG levels, but this does not fully explain her symptoms or the noted vasculitic features.

After correlating her entire history, biopsy results, blood investigations, and examinations over the past five years, and linking these findings to her COVID-19 infection, an extended MDT involving microbiology, rheumatology, ENT, and vasculitis specialists concluded that strongest differentials are atypical vasculitis, immunological response, or an autoimmune condition post-COVID with a very unusual presentation. She was discharged with ENT outpatient follow-up and a positron emission tomography-computed tomography (PET-CT) scan, which came back normal.

While the literature includes cases of COVID-19-induced vasculitis, this specific presentation has not been previously reported. A case report by Allez et al.highlighted a case of COVID-19-related IgA vasculitis in a 24-year-old male presenting with skin rash, intense asymmetric arthralgia, periarticular swelling, and abdominal pain [2]. Kitamoto et al. reported newly diagnosed ANCA-associated vasculitis after COVID-19 infection; however, this report highlighted a presentation with fever and cough but with no pharyngeal involvement at all [3]. In 2023, Acosta et al. published a case report describing a patient with progressively worsening cutaneous lesions over the preceding four weeks [4].​​​​​​​ Frasier et al. highlighted secondary vasculitis post-COVID-19, but none of the 27 cases mentioned in the article had that novel diagnosis [5]. Galeotti and Bayry reported that COVID-19 can trigger autoimmune responses through molecular mimicry, bystander activation, and epitope spreading, leading to conditions such as vasculitis and autoimmune cytopenias [6]. Another case of ANCA vasculitis following COVID-19 syndrome was published by Morris et al., who reported a patient presenting with recurrent cough, hemoptysis, and central chest pain that had started two weeks prior, along with progressively worsening cough and fatigue [7]. Muscle pain and loss of appetite were also noted. The patient denied fever, shortness of breath, nausea, vomiting, and/or diarrhea at the time of presentation, but never had any sort of oropharyngeal lesions [7]. Another case-based review by Suszek et al. described IgA vasculitis following COVID-19, with presentation including respiratory tract infection, dry cough, and subfebrile states [8]. A case report by Hakroush and Tampe described ANCA-associated vasculitis presenting with rhabdomyolysis and pauci-immune crescentic glomerulonephritis following Pfizer-BioNTech COVID-19 mRNA vaccination [9]. In the Wiley Online Library, a case report and literature review were conducted from December 1, 2019, to July 14, 2023, using the keywords “anti-neutrophil cytoplasmic antibody-associated vasculitis” and “COVID-19.” A total of 46 case reports were reviewed, which included presenting complaints of neuropathy, but none reported oropharyngeal lesions [10]. Wnuk et al. published a case report and literature review on COVID-19 and vasculitis. The patient presented with dyspnea, fatigue, and persistent weight loss over the month before hospitalization [11]. Another case of ANCA-associated vasculitis in a 16-year-old female following SARS-CoV-2 infection, along with a systematic review of the literature, was published in BMC in 2022. The presentation included cough, wheezing, hearing loss, arthralgias, and rash [12]. COVID-19 vasculitis and novel vasculitis mimics were reported by McGonagle et al., identifying various presentations; however, the novel presentation in our case was not observed in their report [13]. In 2022, Özeri̇K et al. reported cases that included shortness of breath, highlighting the novelty of our presentation [14]. Two case reports by Chomičienė et al. in 2022 described COVID-19 immunization-induced skin vasculitis presenting with urticaria, angioedema, and rash [15].

## Conclusions

This patient presented with severe symptoms, including pharyngeal hemorrhage, complicated by systemic involvement such as PE and recurrent infections. These challenges made both diagnosis and treatment difficult. A multidisciplinary approach involving ENT, rheumatology, immunology, and infectious disease teams was essential. Her poor response to steroids and antifungal medications highlights the importance of alternative immunosuppressive therapies, such as IVIG or rituximab, to manage atypical vasculitis.

This case represents an unusual and complex presentation of atypical vasculitis, immunological response, or an autoimmune condition following COVID-19. The recurrent oropharyngeal and pharyngeal lesions and systemic involvement support these possibilities, although the exact etiology remains unclear. The case demonstrates the importance of an integrated, multidisciplinary approach in diagnosing and managing post-COVID-19 complications. Further research is still needed to better understand the mechanisms underlying these conditions and develop effective treatments.
